# Structure and distribution of chalky deposits in the Pacific oyster using x-ray computed tomography (CT)

**DOI:** 10.1038/s41598-020-68726-4

**Published:** 2020-07-21

**Authors:** Roxanne M. W. Banker, Dawn Y. Sumner

**Affiliations:** Department of Earth and Planetary Sciences, University of California Davis, Davis, CA 94582 USA

**Keywords:** Marine biology, X-ray tomography

## Abstract

Oysters are unusual among bivalves in that they possess chambers, often filled with soft, chalky calcite, that are irregularly scattered throughout the shell. Because the function of these so-called chalky deposits is still unclear, evaluating the growth and distribution of chalk is important for elucidating the ecological function of this unique shell trait. Specimens of the Pacific oyster *Magallana gigas*, an oyster well known for chalk expression, were grown in Bodega Harbor, Bodega Bay, CA. At the end of an 11 month growing period, specimens were culled and selected animals were submitted for x-ray computed-tomography imaging. Three-dimensional reconstructions of oyster shells were used to assess the overall distribution of chalk, and also to better understand the relationship between chalk and other structures within the shell. Results indicate that chalky deposits underly sculptural features on the shell exterior, such as external ridges and changes in growth direction, and also that there is a relationship between chalk formation and oyster processes of cementation. Overall, chalk is useful for a cementing lifestyle because it enables morphological plasticity needed to conform to irregular substrates, but also acts as a cheap building material to facilitate rapid growth.

## Introduction

Many researchers have used preserved remains of shells (molluscan or otherwise) to improve scientific understanding of paleoecosystems and organismal interactions in the fossil record^1–3^. Oysters (Bivalvia: Ostreidae) have utility in this setting because they are well preserved in the fossil record, and are widely geographically distributed in paleo and modern ecosystems^4^. Moreover, stable isotope and elemental records derived from bivalve shell carbonate reflects the environmental conditions in which the shell grew^5–9^. These geochemical datasets have been used to develop proxies, reconstruct paleoclimate, and characterize the environment in which fossil oysters lived (e.g. estuarine versus fully marine)^10–13^. Stable isotopes have also been used to assess growth rate, season of death, and the occurrence of phytoplankton blooms in both modern and fossil Ostreids^14–16^.

Oyster shells are composed primarily of foliated calcite interspersed with lens shaped chambers^17,18^. These chambers are irregularly interspersed throughout the shell and are often filled with porous calcite, also referred to as chalk or chalk deposits. It is important to note that chalk is distinct from the vesicular calcite formed by some members of Gryphaeidae (Ostreoidea), which consists of hollow columnar spaces composed of granular calcite, as opposed to the interconnected calcite laths that are characteristic of chalk^19,20^. Chambers and chalky deposits are found in both extant and fossil oysters, though the function of these features, particularly chalk and how it forms, has been the subject of much scientific inquiry (e.g.^21–23^). Ontogenetically, the formation of chalk commences after the transition to the dissoconch stage when the oyster settles as spat, and continues throughout adulthood^24^. Numerous authors have hypothesized about the mechanism of chalk formation. While some studies have proposed that chalk is precipitated by the animal itself as regulated by the mantle^25–27^, others have suggested that the unique crystal structure of this feature indicates that it is a product of microbial mineralization within the oyster shell^18,23,28^. Finally, recent work suggests that chalk is formed when the mantle pulls away from previously deposited shell material more quickly than for folia, resulting in the porous microstructure of chalk^20^. Overall, differences between the formation and initiation of chalk instead of foliated calcite remains unclear. A more complete understanding of how this shell feature precipitates could also inform interpretations of fossil record and geochemical archives of oyster shells. For example, if chalk and folia are not precipitated simultaneously by oysters, then proxy data taken from only folia would not necessarily represent a continuous record.

The goal of the present research is to further characterize the growth and structure of chalky deposits in *Magallana gigas* (also known as *Crassostrea gigas*), the Pacific oyster, and to use these data to better understand the ecological function of this trait in oysters. A cohort of oysters was reared in Bodega Bay from June 2016 to May 2017. Select individuals, the twenty largest oysters that also possessed a shell shape typical of the cohort, were sacrificed, and shells were imaged using a micro X-Ray Computed Tomography (CT) scanner, which provided high-resolution 3D visualization of shells, chambers, and chalk.

## Results

### Visual description of shells

**Figure 1:**
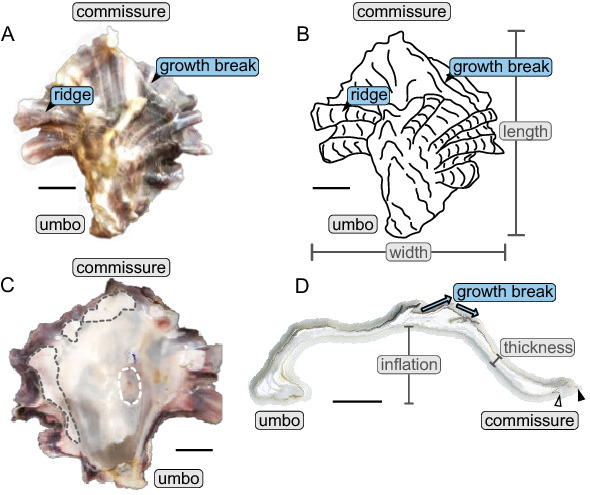
Example of *M. gigas* specimen sampled for this study (CG1-A01). (**A**) Exterior view of left valve, and (**B**) line schematic of the same valve. The same ridge and growth break is indicated in panels (**A** and** B**), correlating these features between the two views of the shell. (**C**) Interior view of the same valve, where chalky deposits are outlined in a dashed gray line, and the muscle scar is outlined in a dashed white line. (**D**) Cross section from an oyster belonging to a different cohort of oysters grown under the same conditions. The gray halo around the shell is the epoxy used to mount and section the shell onto a glass slide. Chalk appears as opaque white lenses interspersed throughout the shell, whereas the folia present as translucent to gray in color. The newest layer of folia at the commissure (open triangle) does not extend past the previous layer (filled triangle). These structures together represent an incipient growth break. Scale bars are 1 cm.

Like all *M. gigas*, shells from oysters grown in Bodega Harbor possess two oval calcareous valves of unequal convexity. The left cementing valves are generally deeply convex (i.e. cup shaped with high inflation), whereas the right non-cementing valves are flat or planar (Fig. 1). The two valves are hinged dorsally near the characteristic beak, or umbo, on the left valve. The valves are held together by an organic ligament as well as articulating teeth on the hinge, which are present on the interior surface of both valves. The ventral margin, or commissure, is able to open freely, and each valve tapers to a fine edge before transitioning to a very thin layer of periostracum, the outermost organic layer of the shell.

Accretionary growth of *M. gigas* proceeds with the addition of material from the umbo towards the commissure, and shell thickening is achieved by addition of material to the interior shell surface. Conspicuous on the exterior of shells are radial ridges, hereafter referred to as ridges, that trend from the umbo to the commissure. These ridges may extend to the commissure itself, but may terminate closer to the umbo. The exterior surfaces of oyster shells possess concentric laminae, which represent the lateral growth of the organism from the umbo towards the commissure. Some of these laminae terminate abruptly in terrace-like features on the shell, marking a transition from outward growth to growth that is oblique to the previous growing plane (i.e. more inward growth), resulting in higher inflation and a more deeply cupped shape for left valves (Fig. 1). These areas where growth direction changes abruptly will hereafter be referred to as growth breaks. Chalky deposits occur from just inside the commissure to the umbonal region, and vary widely in size and shape (Fig. 1). Also present on the interior surface of both valves is the oval adductor muscle scar.

**Figure 2:**
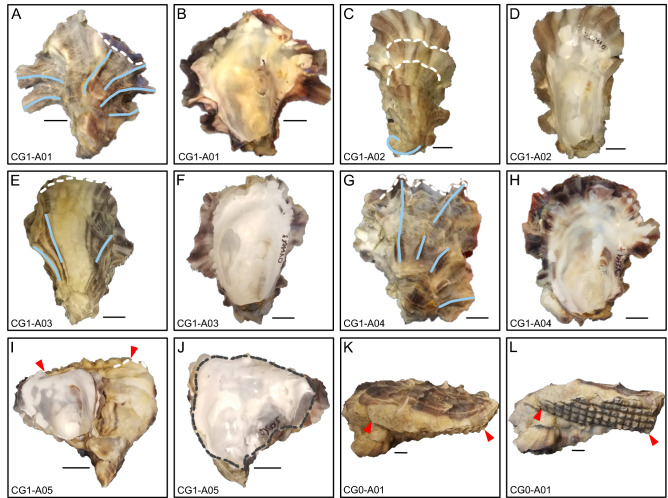
Panels (**A**)–(**J**) show the exterior and interior view of the left valve of each of the five oyster specimens submitted for CT-scanning in this study, and the specimen ID is indicated in the bottom left-hand corner of each panel. White dashed lines show the position of major growth breaks for specimens that have them. Though the white dashed line on specimens CG1-A03, CG1-A04, and CG1-A05 appears to occur at the commissure, this actually represents a growth break where additional shell material is not viewable from the angle of the photographs. Ridges are highlighted by blue solid lines, however, in panel (**C**), the solid blue line outlines the crescent shaped umbo of CG1-A02. The red markers in panels (**I**), (**K**), and (**L**) demarcates the extent of the cage mesh pattern imprinted on shells that recemented onto cage material. The gray dashed line in panel (J) denotes the irregularly shaped commissural margin of that specimen. The oyster displayed in panels (**K**) and (**L**) was grown in a separate cohort of oysters and was not submitted for CT-scanning because it was too large for the scanner. Panel K shows the exterior view of the left valve of specimen CG0-A01, and panel L was taken at an oblique angle to better display the waffled pattern on the shell. Scale bars are 1 cm.

### Shape of typical shells

*M. gigas* specimens CG1-A01, CG1-A02, CG1-A03, and CG1-A04 were chosen for CT imaging because they possessed a shell shape and other morphological characteristics that were commonly observed in the oysters grown in Bodega Harbor (Fig. 2). On average, shells submitted for scanning were 59.0 ± 6.2 mm (n = 5) from umbo to commissure (i.e. shell length), and 46.4 ± 8.1 mm wide (n = 5), where width was measured at the widest point perpendicular to the shell length. Inflation of shells, measured as the maximum distance left valve and the hinge line between the umbo and the commissure, averages 14.2 ± 3.1 mm (n = 5).

Valves were approximately 2–3 mm thick near the commissure before tapering to a fine edge, but were thicker towards the umbo, which was 5–6 mm thick in most specimens. The number and size of ridges expressed on shell exteriors varies widely amongst the oysters. For example, CG1-A01 displays six ridges, 1–2 mm in width, that are easily identifiable on the shell surface (Fig. 2A,B). In contrast, CG1-A02 does not have any ridges that contact underlying shell layers, but has pronounced growth breaks where the overhanging shell layer terminates in ridges (Fig. 2C,D). CG1-A03 has just three ridges on the shell exterior, and only has distinct growth breaks near the commissure (Fig. 2E,F). Ridges within a single specimen also vary in length, and may extend over the full length of the shell, or end closer towards the umbo. Changes in growth direction occur both as growth breaks, which result in higher inflation of valves, and as a change in the primary growth direction of the valve. The latter phenomenon yields a curved shell shape, which is exemplified by specimen CG1-A02 that has a crescent shaped umbo, indicating a change in growth direction early in ontogeny (Fig. 2C).

**Figure 3:**
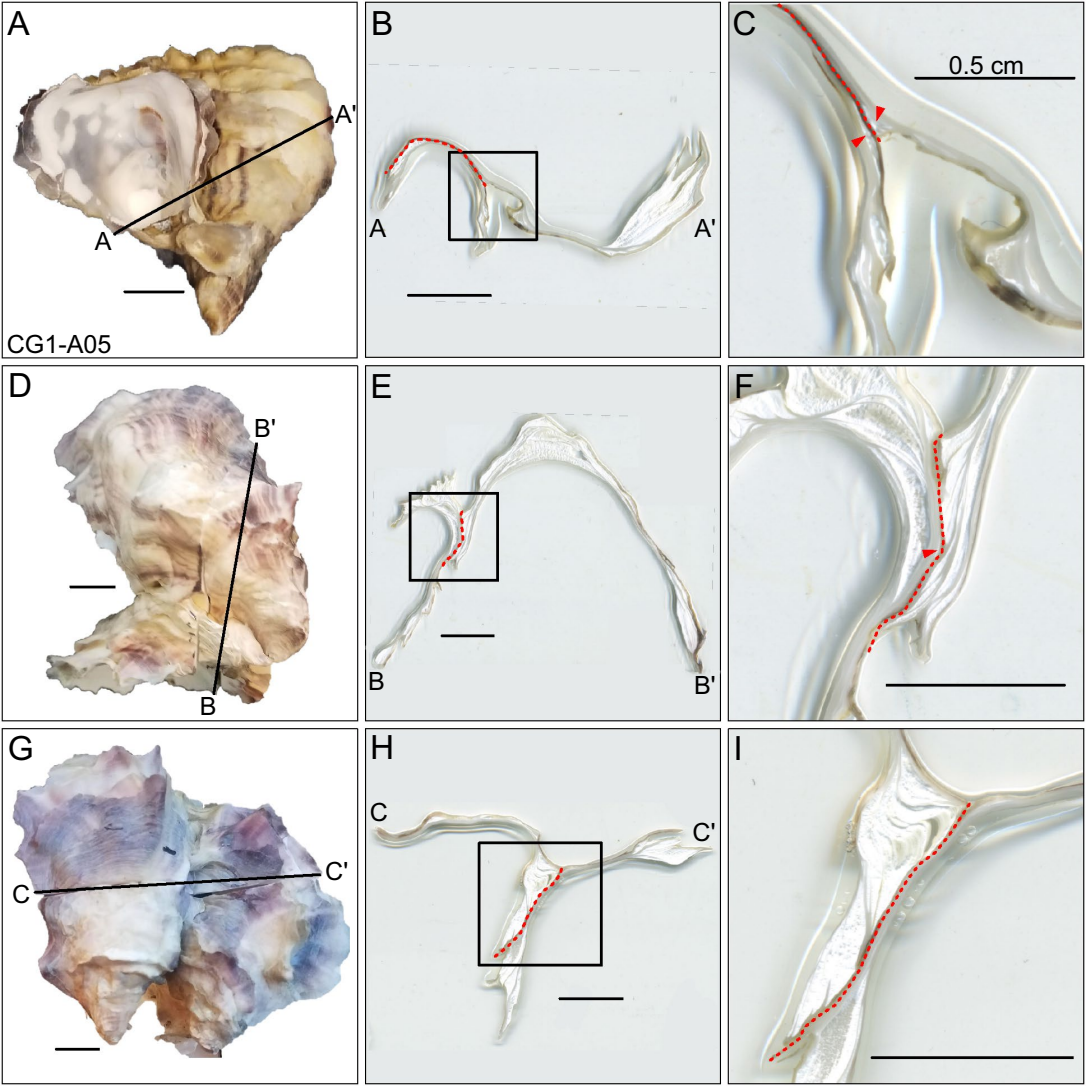
Cross sections through suture zones of pairs of oysters that recemented to one another. Panel (**A**) depicts the exterior view of CG1-A05, with the position of the cross section in (**B**) indicated by the black line from (**A** to** A′**). Panel (**C**) is magnified from the inset in (**B**). Panels (**D**), (**E**), and (**F**) show the exterior view, cross section (**B** to** B**′), and magnified inset of another set of oysters that recemented to one another. Panels (**G**) through (**I**) are from a third set of recemented oysters (**C** to** C**′). Red dashed lines indicate the area of attachment between two individuals. Red arrows point to small chalky deposits that are located near this border, but are still found within the periostracum of an individual. Scale bars are 1 cm unless otherwise noted.

### Shape of recemented shells

Shells that recemented onto a hard substrate, such as another oyster or cage mesh material, display much greater variability in shell morphology than their counterparts that remained unattached throughout ontogeny. The commissures of animals that recemented are still generally ovate, but may also possess outgrowths that make the shell margin outline highly irregular (Fig. 2J). In addition, while the commissure of specimens CG1-A01 through CG1-A04 lie primarily in the same plane, the shell margin of CG1-A05 does not and the contour of the commissural margin may be convex or concave. Finally, recemented shells tend to retain the shape of the object onto which they were growing. For example, CG1-A05 retains the waffle-like pattern of the cage mesh onto which it had recemented (Fig. 2I). This texture, a result of growing onto cage mesh, can also be seen in specimen CG0-A01 (Fig. 2K,L). Though CG0-A01 was not submitted for CT scanning, it represents an excellent example of the morphological plasticity exhibited by specimens that grew onto another substrate. Cross sections of the suture zones of recemented oysters, specimen CG1-005 and two additional pairs of animals, showed that while chalk was abundant where oysters grew onto one another, there was no chalk laid down outside of an individuals’ periostracum (Fig. 3).

### 3D visualization of shells

**Figure 4:**
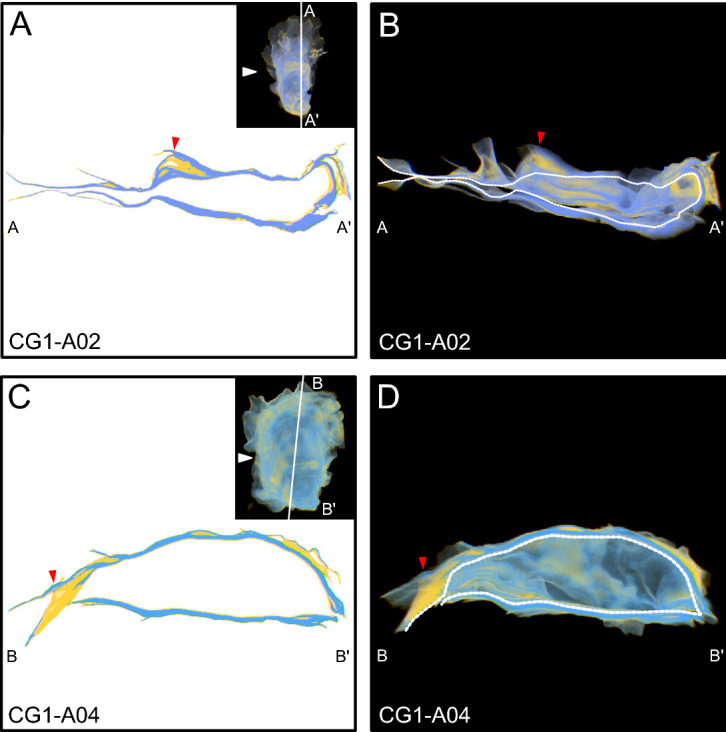
View of specimens as observed in 3D-Visualizer used to exemplify chalk as it relates to growth breaks. For each specimen, a cross section view is shown (left) and a cut-away of the 3D-reconstruction, which shows the cross section, but also has the rest of the shell rendered in 3D in the background (right). The thumbnail in the cross section panels shows the entire 3D reconstruction of the specimen, and the white line indicates position of the cross section and cut-away in the shell. The white triangle in the thumbnail indicates the point of view of the cutaway. The white-dashed lines in cut-away panels demarcates the separation between the cutting plane and the interior of the specimen in the background. For each image, chalk and folia are indicated by yellow and blue, respectively. Red arrows correlate specific features between the cross sectional views on the left and the 3D reconstructions on the right. For this figure, red arrows also highlight chalky deposits found under significant growth breaks.

**Figure 5:**
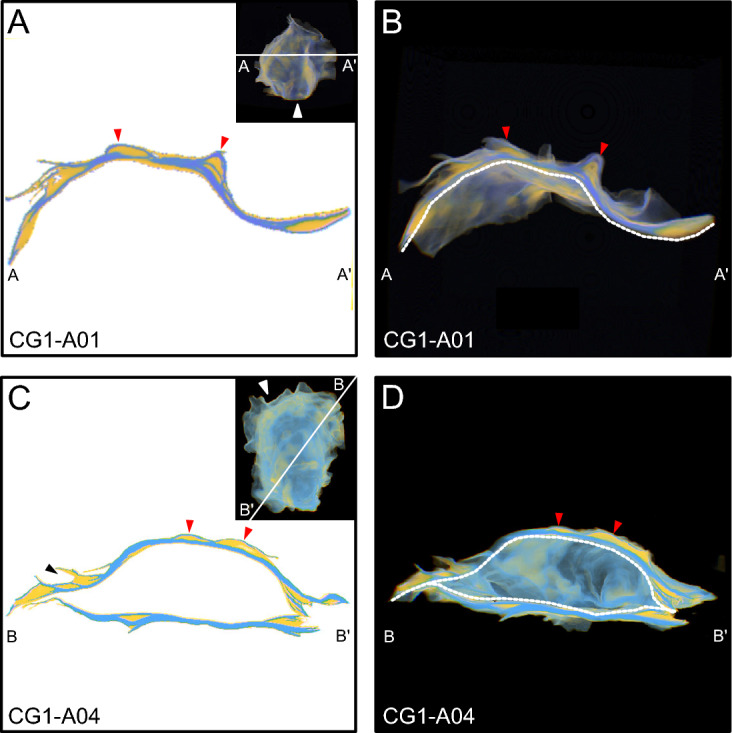
View of specimens as observed in 3D-visualizer used to exemplify chalk as it relates to external radial ridges. See Fig. 4 for a description of the individual panels. Here, red arrows indicate the position of chalky deposits under external radial ridges. The black arrow in C indicates a small chalky deposit near the umbo that was not completely sealed by folia, and remains exposed to the exterior environment.

**Figure 6:**
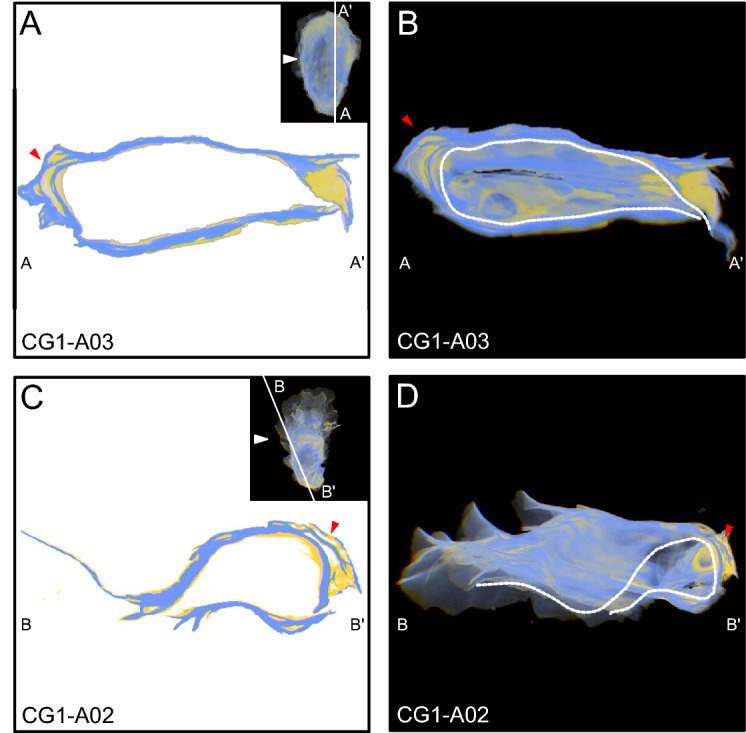
View of specimens as observed in 3D-visualizer used to exemplify chalk as it relates to the curved interiors of umbos. See Fig. 4 for a description of the individual panels. Here, red arrows highlight the position of chalky deposits located on the interior concave surfaces of umbos.

**Figure 7:**
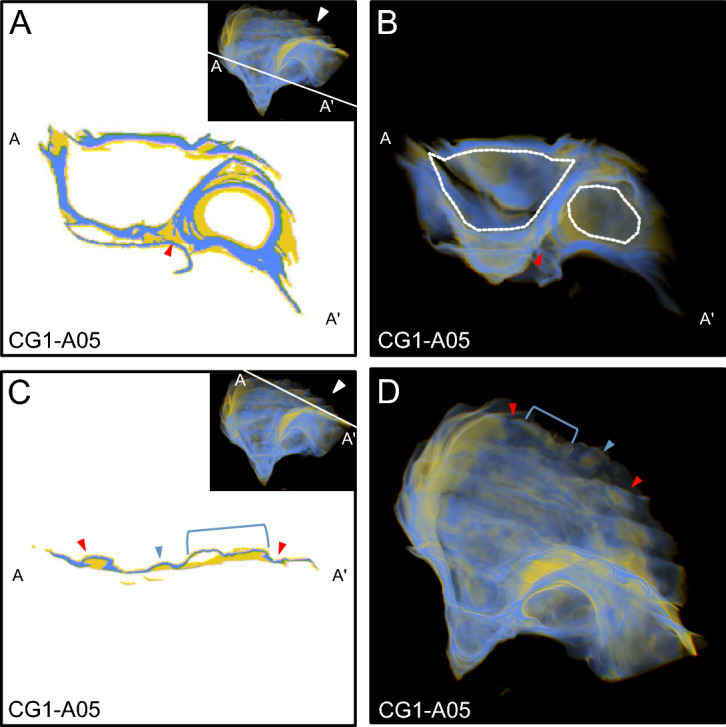
View of specimens as observed in 3D-visualizer used to exemplify chalk as it relates to cementation. See Fig. 4 for a description of the individual panels. Here, panel (**D**) does not have a cut-away image of the same cross-section shown in (**C**), but instead an enlarged image of the thumbnail. Red arrows indicate the terminal ends of the cross section and correlate features between panels (**C**) and (**D**). The blue arrow and blue bracket correlate the presence of specific chalky deposits between both panels.

The calcitic folia are a major component of these oyster shells, and define the structure of the external features. For example, folia define growth breaks (Fig. 4), and ridges are supported by complex structures composed of folia, creating the sculptures present on the shell exterior (Fig. 5). Three-dimensional reconstructions of oyster shells revealed that chalky deposits are expressed throughout the shell from umbo to commissure (Figs. 4, 5, 6, 7). Smaller chalky deposits are present irregularly throughout shells, including on the interior shell surface. Significant chalk formation occurs in the umbo (Fig. 6), beneath ridges (Fig. 5), and underlying growth breaks (Fig. 4) and other changes in growth direction, such as the curved umbo in CG1-A02. Chalky deposits in the umbo are generally lens shaped and are fully surrounded by folia, apparently sealing them within the shell (Fig. 6).

**Figure 8:**
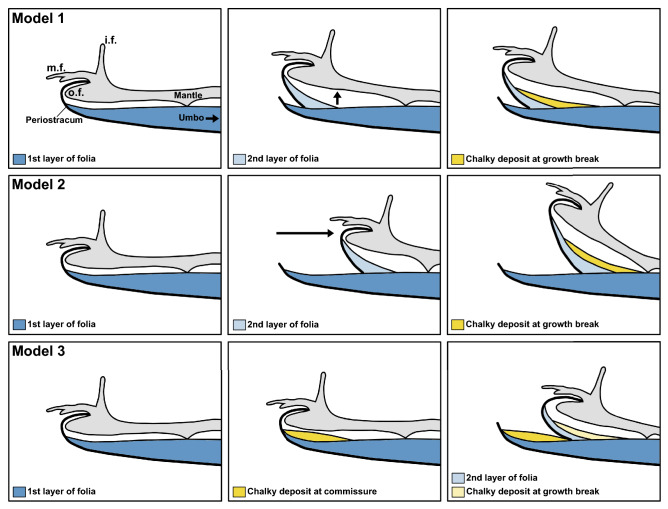
Schematic representation of three possible models for growth break formation on the left valves of oysters. i.f.: inner fold of the mantle; m.f.: middle fold of the mantle; o.f.: outer fold of the mantle. The periostracum is represented by the thick black line, originating from between the middle and outer mantle folds. Model 1 (row 1): First, the mantle lays down the periostracum, followed by an initial layer of folia. Next, the mantle separates from the previously formed shell (black arrow), and without retracting, begins forming a new layer of periostracum and folia, creating a growth break. Last, a chalky deposit is formed in the interior depression resulting from the previous two steps. Model 2 (row 2): First, the mantle lays down the periostracum and the initial layer of folia. Next, the mantle retracts (black arrow), and forms a new layer of periostracum followed by the next layer of folia. Last, chalk is precipitated in the interior depression resulting from the previous two steps. Model 3 (row 3): First, the mantle lays down the periostracum and the initial layer of folia. Next, a small chalky deposit forms near the commissure. The new layer of periostracum and folia is formed at an angle oblique to the previous growing plane because of the presence of the chalky deposit. Last, another chalky deposit is formed on the interior depression resulting from the previous two steps. Schematic of shell and mantle adapted from Yamaguchi^32^.

## Discussion

*Morphology and distribution of chalk and folia* The timing of chalk deposition in relation to folia precipitation has been a long standing question in regards to chalky deposit formation. For bivalves in general it is well recognized that elongation is achieved by the addition of new material in the spiral direction (i.e. to the commissural margin), and shell thickening occurs by the addition of carbonate to the interior shell surface, beneath (i.e. interior to) old shell material^17^. Chalky deposits observed in 3D reconstructions were found interior and ventral to folia, and most were completely sealed within the shell by surrounding folia. This was true for chalk found in the umbo, as well as chalky deposits that formed directly beneath growth breaks (Figs. 4,6), though it remains unclear if calcification continues in chambers after they are sealed, as has been hypothesized previously^23^. Although not common, examination of 3D reconstructions also revealed the presence of chalky deposits that were not completely sealed within the shell. These appear to be the result of a small amount of chalk that was precipitated onto folia at the commissure, which was not completely sealed when the next layer of folia was deposited, leaving a small wedge of chalk exposed to the shell exterior (Fig. 5C). The position of folia as exterior to chalk indicates that folia, not chalk, is precipitated as the outermost shell layer that underlies the periostracum, and consequently provides structural support to the shell, as has been noted in previous work^29^. In addition, the deposition of folia produces external features, such as ridges, growth breaks, and other changes in growth direction, thus creating depressions on the interior shell surface that are subsequently filled with chalk. The pattern of chalk formation beneath growth breaks and other external features confirms previous observations that chalk fills in depressions on the interior surfaces of shells^18,20,24,26,27,30,31^, but also provides context for how these depressions form.

For oysters, growth breaks represent a significant change in growth direction, and are important in the context of chalk formation because these features are often associated with chalky deposits. Therefore, a more complete understanding of how growth breaks form can help elucidate the function and timing of chalk growth. Based on the observations regarding the relationship between chalk and folia, there are three possible models for shell formation at growth breaks, which all have different implications for the function of chalk in oyster shells, and also the behavior of the mantle during shell formation more generally (Fig. 8).

*Model 1* First, the mantle lays down the periostracum (the outermost organic layer of the shell) which is followed by deposition of a thin layer of folia directly beneath, towards the shell interior. Next, the mantle separates from the previously formed shell, and without retracting significantly, begins forming a layer of periostracum and folia at an oblique angle to the previous growing plane, creating a growth break. This new geometry creates a depression on the interior shell surface, directly below where the second layer of periostracum and folia branched off from the first. This depression is subsequently filled with chalk. As the oyster continues to thicken and extend its shell outward, this chalky deposit beneath the growth break is sealed within the shell with a new layer of folia. In Model 1, the growth break is caused by the mantle detaching from the initial layer of periostracum, which may be caused by breakage of the periostracum at the commissure, or some other interruption (Fig. 8: row 1).

*Model 2* First, the mantle lays down the periostracum, which is followed by deposition of a thin layer of folia directly beneath the external organic layer. Next, the mantle detaches from the currently forming layer of periostracum and retracts to a point along the previously formed shell. Then, the mantle begins forming a new layer of periostracum and folia, inward from the previous layer, at an angle oblique to the previously growing plane. This new geometry creates a depression on the interior shell surface, directly interior to where the second layer of periostracum and folia branched off from the first. This depression is subsequently filled with chalk. As the oyster continues to thicken and extend its shell outward, this chalky deposit beneath the growth break is sealed within the shell by a new layer of folia. In Model 2, the growth break is also caused by the mantle detaching from the initial layer of periostracum, which may be caused by breakage of the periostracum at the commissure, or some other environmental interruption. This scenario differs from Model 1 in the behavior of the mantle. In Model 2, the mantle retracts and builds new shell material inward from the previous growing plane. In contrast, in Model 1, the mantle does not retract; rather, it moves toward the interior space between valves and precipitates the next layer while remaining extended (Fig. 8: row 2).

*Model 3* First, the mantle lays down the periostracum, which is followed by deposition of a thin layer of folia directly beneath the external organic layer. Next, the mantle precipitates a small mound of chalk that extends to the outermost extent of the previously grown shell. The mantle detaches from the currently forming layer of periostracum and retracts to a point along the previously formed shell. Then, a new layer of periostracum and folia is precipitated interior to the initial chalk mound, and extends past this chalky deposit, thus leaving the chalk exposed to the shell exterior. This new growth break also creates a depression on the interior shell surface, directly below the point where the first folia, the umbo end of the chalk mound, and the second layer of folia and periostracum intersect. This space is subsequently filled with chalk, and as the oyster continues to thicken and extend its shell outward, the chalky deposit beneath the growth break is sealed within the shell. Here, the formation of the growth break is not driven by the separation of the mantle from the first layer of periostracum. Instead, the change in growth direction is a result of the formation of the chalk mound, which directs new growth at an angle oblique to the previous growing plane. For shells observed in this study, growth breaks are not associated with chalky deposits (i.e. the initial chalk mound described here) that are exposed to the shell exterior, as in Model 3. Therefore, we can exclude Model 3 as an explanation for growth breaks, and the change in shell growth direction immediately following a growth break (Fig. 8: row 3).

Models 1 and 2 are more difficult to differentiate based on shell morphology alone because as described, they result in the same, or very similar, set of features, and the major differences between these two models concerns the behavior of the mantle during growth break formation. However, other lines of evidence can be used to parse out which model is more likely the to produce the shell features observed. For Model 1, the mantle does not retract and instead moves away from the previous growing plane by pivoting away from the old shell. Therefore, the new layer of periostracum and folia should be the same length as the initial layers, because the mantle has remained fully extended. In contrast, if the mantle retracts (Model 2) and builds the second layer of periostracum outward from old shell material, the newer layers will be shorter (i.e. terminate closer to the umbo than the first layer of folia) until enough growth has occurred to extend new folia past the old shell material. This second pattern has been observed in the cross section in Fig. 1D. Therefore, Model 2, which includes mantle retraction, is the most likely representation of how growth breaks form and how the mantle behaves in this context. In addition, Model 2 agrees well with previous work in which oyster mantles were directly observed to contract during shell formation and cementation^32^. In an experiment in which the shell margin of the left valve of an oyster was removed, the mantle subsequently began forming a new layer of periostracum on old shell material, dorsal to the commissure (^32^, Fig. 7B). This illustrates that the mantle does retract in order to form new periostracum and shell outward from old shell material consistent with Model 2. Additional observational work, such as that performed by Yamaguchi^32^, coupled with careful measurements to establish how far back the mantle retracts during growth break formation, would yield definitive evidence to support Model 1 or Model 2.

*Chalky deposits and cementation* Chalky deposits were observed to be extensive at sites where shells reattached to a substrate, such as where oysters cemented to other individuals or cage mesh material (Figs 2L, 7). For oysters that recemented onto cage material, the periostracum was the outermost layer, indicating it was laid down first to conform to the irregular surface. Similar observations were also made by Yamaguchi^32^, who also proposed that the mantle is an active participant in shell cementation by pressing newly made periostracum into the substrate, thus promoting attachment. Therefore, chalky deposits are involved in cementation at least to the extent that chalk allows the oyster to conform to an uneven substrate while maintaining a favorable internal space.

Interestingly, Harper^33^ recorded the presence of a chalky deposit outside of an *M. gigas* shell, between the periostracum and the substrate. If the presence of chalk between the shell and the substrate is a common occurrence, then this would indicate that chalk plays a more central role in cementation than simply conferring the ability to conform to an uneven substrate. In the present study, three pairs of oysters that had recemented to one another were sectioned and examined for these chalky deposits occurring outside of the periostraca (Fig. 3). However, none were found. Although this may indicate that a larger sample size is required to survey for this phenomenon, we propose that external chalky deposits, such as that found by Harper^33^, were aberrant. This indicates that chalk is generally not directly used for oyster attachment. But, how was the chalk observed by Harper^33^ emplaced between the periostracum and the substrate?

While the mantle pressing of the periostracum to the substrate may be one component of the cementation process for *M. gigas*^32^, a mantle-produced organic-inorganic adhesive plays a central role in attaching oysters to the substrate. Harper^33^ hypothesized that the organic fraction of the cement is able to move through the permeable periostracum to fill the space between this layer and the substratum, thus attaching the oyster. There is additional evidence to suggest that mantle secreted organics play a central role in determining crystal morphology in molluscan shells^34–39^. Thus, if the mantle-secreted organic precursors to chalky calcite were inadvertently leaked through the porous periostracum, this may explain the present of chalk in a space that would normally be occupied by cement^33^. While this remains a speculative hypothesis, additional work characterizing the organic constituents of shell layers, and addressing the ability of these organics to mobilize through the periostracum, would help to resolve these questions.

*Functional ecology of chalk* Data presented here indicate that chalk is associated with morphological variability in *M. gigas*, and is useful for cementation because it allows the animal to conform to an irregular substrate while maintaining a favorable internal space. Although many other bivalves are able to achieve a cementing lifestyle without the use of chalk, the high porosity of chalk produces a shell structure that is much less dense than surrounding folia^19,40^. In addition, chalk formation is associated with rapid growth rates, and very large, thick shells^10,23^. This indicates that chalk, in addition to being useful for cementation and accommodating morphologic plasticity, enables rapid shell growth by acting as a relatively cheap building material during shell construction. Future work comparing the strength or durability of cementation in oysters that produce chalk versus oysters and other cementing bivalves that do not (e.g. Chamids) would provide additional insight into the tradeoffs associated with using this material to aid cementation. Last, although recent work suggests that folia and chalk are precipitated synchronously^20,41^, open questions remain regarding the timing of folia versus chalk deposition within the shell. Additional experiments employing both marking techniques (e.g. calcein) and stable isotope geochemistry would help to determine the temporal relationship between chalk and folia, particularly as it relates to the formation of growth breaks and other features discussed here.

## Methods

### Study organism, oyster culturing, and environmental setting

For this study, chalky deposit formation was investigated in *M. gigas*, which is commonly farmed on the coast of California. All specimens of *M. gigas* obtained for this experiment were spawned by by Hawaiian Shellfish LLC. In nature, oyster larvae undergo a metamorphosis during which they settle to the seafloor as spat (juveniles) and cement themselves to a hard substrate, which may either be a rocky substrate or another oyster^42^. Aquaculture practices regularly sort and separate juvenile oysters by size, yielding singles (i.e. individual oysters that are unattached to other individuals) that are suitable for commercial purposes. When settled juveniles reached a size of 1.4 mm (+/− 0.2 mm) they were placed on ice and shipped overnight to the Starbird Mariculture, Inc facility in Bodega Harbor, Bodega Bay, CA (38$^{\circ }$19’41.56”N, 123$^{\circ }$03′22.61″W). Oysters were grown in the Floating Commercial Upweller System (FLUPSY) at Starbird Mariculture Inc. until they reached a size of 4–5 mm (approximately 4–7 months old). At this time, oysters used for this experiment were separated and moved to mesh cages that were secured to a dock in Bodega Harbor immediately adjacent to the FLUPSY and all specimens were initially singles (i.e. unattached to other individuals). Oysters were monitored and cleaned of epibionts approximately every 2–3 weeks. The permits necessary for outplanting oysters and using the following protocols were obtained from the California Department of Fish and Wildlife.

Bodega Harbor is a relatively shallow bay on the California coast that is protected from wave action by two parallel jetties at its entrance. The harbor is well ventilated and is almost completely flushed during each tidal cycle. Bodega Harbor receives little freshwater input from April to November, and is periodically exposed to nutrient rich water during strong upwelling events^43^.

### Computed tomography

Oyster specimens that were submitted for computed tomography (CT) scanning were moved from the FLUPSY to synthetic mesh cages on June 19, 2016. The five specimens listed in Table 1 were sacrificed on May 5, 2017 and submitted for CT analysis thereafter. Specimens CG1-001 through CG1-004 were singles that were selected because they displayed shell morphology that was representative of the rest of the population. Over the course of this experiment it became apparent that oysters were able to secondarily cement to a hard substrate long after the initial settlement phase, including to other oysters and the cages themselves. CG1-005 was selected as an example of two singles that cemented onto one another and cage material, yielding a specimen with two individuals. A separate oyster cohort was outplanted prior to the primary group for a separate experiment and reared under the same conditions until animals were culled. One specimen from this group, CG0-A01, was used for the present study because it also exemplified the effect of cementation on shell morphology. However, this specimen was not submitted for CT-scanning because it was too large for the scanner.

**Table 1:** Table of oyster specimens submitted for CT scanning and their measurements (mm) as shown in Fig. 1.

Specimen ID	Width	Length	Inflation
CG1-A01	58.3	59.4	14.8
CG1-A02	39.6	66.3	15.1
CG1-A03	41.9	61.4	17.6
CG1-A04	51.3	58.6	14.5
CG1-A05	42.0	49.2	9.1

X-ray tomographic images were obtained on the Zeiss Xradia 520 Versa (Carl Zeiss Microscopy, Inc.) with the Flat Panel extension at the UC Davis Center for Molecular and Genomic Imaging (CMGI). This produced 46 um x 46 um x 46 um voxels. Center for Active Visualization in the Earth Sciences (http://KeckCAVES.org) open source software for 3D visualization was used to view scans, specifically Vrui^44^ and 3DVisualizer^45^.

### Recemented shell cross sections

To characterize attachment of oysters that recemented to one another (i.e. non-singles), specimen CG1-005 and two additional oysters randomly selected from the same cohort that were not submitted for CT scanning were sectioned through the suture zone between two individuals. This was done using a Buehler IsoMet low-speed saw. The cut surface was ground using a 600 grit diamond wheel, and the shell-half was mounted onto a large slide (51mm × 75mm) using Hillquist AB thin section epoxy. Shells were then re-sectioned using Buehler PetroThin thin section saw, resulting in mounted shell sections that were approximately 500–700 $\mu$m thick. Sections were digitized using a scanner.

## Data Availability

Computed tomography scan files are available in .txm format from Dryad (10.25338/B8X02M).
